# Molecular Tweezers Targeting Transthyretin Amyloidosis

**DOI:** 10.1007/s13311-013-0256-8

**Published:** 2014-01-24

**Authors:** Nelson Ferreira, Alda Pereira-Henriques, Aida Attar, Frank-Gerrit Klärner, Thomas Schrader, Gal Bitan, Luís Gales, Maria João Saraiva, Maria Rosário Almeida

**Affiliations:** 1 IBMC, Instituto de Biologia Molecular e Celular, Universidade do Porto, Rua do Campo Alegre, 823, 4150-180 Porto, Portugal; 2 Department of Neurology, David Geffen School of Medicine, University of California at Los Angeles, Los Angeles, CA USA; 3 Brain Research Institute, University of California at Los Angeles, Los Angeles, CA USA; 4 Faculty of Chemistry, University of Duisburg-Essen, Essen, Germany; 5 Molecular Biology Institute, University of California at Los Angeles, Los Angeles, CA USA; 6 ICBAS, Instituto de Ciências Biomédicas Abel Salazar, Universidade do Porto, Porto, Portugal

**Keywords:** Molecular tweezers, Transthyretin, Amyloid, Familial amyloidotic polyneuropathy

## Abstract

Transthyretin (TTR) amyloidoses comprise a wide spectrum of acquired and hereditary diseases triggered by extracellular deposition of toxic TTR aggregates in various organs. Despite recent advances regarding the elucidation of the molecular mechanisms underlying TTR misfolding and pathogenic self-assembly, there is still no effective therapy for treatment of these fatal disorders. Recently, the “molecular tweezers”, CLR01, has been reported to inhibit self-assembly and toxicity of different amyloidogenic proteins *in vitro*, including TTR, by interfering with hydrophobic and electrostatic interactions known to play an important role in the aggregation process. In addition, CLR01 showed therapeutic effects in animal models of Alzheimer’s disease and Parkinson’s disease. Here, we assessed the ability of CLR01 to modulate TTR misfolding and aggregation in cell culture and in an animal model. In cell culture assays we found that CLR01 inhibited TTR oligomerization in the conditioned medium and alleviated TTR-induced neurotoxicity by redirecting TTR aggregation into the formation of innocuous assemblies. To determine whether CLR01 was effective *in vivo*, we tested the compound in mice expressing TTR V30M, a model of familial amyloidotic polyneuropathy, which recapitulates the main pathological features of the human disease. Immunohistochemical and Western blot analyses showed a significant decrease in TTR burden in the gastrointestinal tract and the peripheral nervous system in mice treated with CLR01, with a concomitant reduction in aggregate-induced endoplasmic reticulum stress response, protein oxidation, and apoptosis. Taken together, our preclinical data suggest that CLR01 is a promising lead compound for development of innovative, disease-modifying therapy for TTR amyloidosis.

**Electronic supplementary material:**

The online version of this article (doi:10.1007/s13311-013-0256-8) contains supplementary material, which is available to authorized users.

## Introduction

Transthyretin (TTR) is a 55-kDa tetramer made of identical subunits of 127 amino acids and contains an extensive β-sheet structure [1]. TTR is synthesized mainly in the liver and the choroid plexus of the brain, and is secreted to the plasma and cerebrospinal fluid, respectively. The transport of thyroxine (T_4_) and retinol are the two most recognized physiological functions of TTR. Structurally, TTR has two identical inner T_4_ binding sites located at the dimer–dimer interface. Although TTR carries about 15 % of total T_4_ in the plasma, more than 99 % of the T_4_-binding sites remain unoccupied [1].

More than 100 TTR single point mutations have been reported, most of them associated with abnormal TTR misfolding and self-assembly into amyloid fibrils that display distinct patterns of organ involvement, age of onset, and clinical course. Substitution of methionine for valine at position 30 of TTR (TTR V30M) results from the most common mutation associated with familial amyloidotic polyneuropathy (FAP), a multivisceral and life-threatening disease affecting predominantly the peripheral (PNS) and autonomic nervous system [2,3]. FAP presents a wide geographic distribution, with the largest populations in Portugal, Japan, and Sweden, and is estimated to affect ∼5000–10,000 patients worldwide [4].

Besides peripheral neuropathy, cardiomyopathy, amyloid deposition in the eye, carpal-tunnel syndrome, or amyloid deposition in the leptomeninges are clinical phenotypes associated with different forms of TTR amyloidosis (amyloidosismutations.com).

Although the involvement of the central nervous system is unusual, some TTR variants (L12P, D18G, A25T, G53E) show significant, or even exclusive, central nervous system pathology, including deposition of mutant TTR within leptomeningeal vessel walls and pia-arachnoid membranes, and oculoleptomeningeal amyloidosis [5–8].

In addition, it is important to emphasize that owing to its high degree of β-sheet structure, wild-type (WT) TTR itself has an inherent tendency to self-assemble into β-sheet-rich amyloid fibrils [1]. Thus, a nonhereditary, age-related form of TTR amyloidosis, senile systemic amyloidosis, is associated with WT TTR amyloid deposition in the heart, causing cardiac dysfunction [9].

Proposed therapies for TTR amyloidosis resemble strategies adopted for other protein misfolding disorders. Those therapies include i) gene silencing to block synthesis of the protein or its precursor; ii) stabilization of the native protein structure; iii) modulation of the aggregation pathway; and iv) clearance by disaggregation of amyloid fibrils [10,11]. Here, we investigated whether CLR01, a lysine-specific “molecular tweezer” recently reported to inhibit aggregation and toxicity of multiple disease-related amyloidogenic proteins, including TTR [12], could modulate TTR assembly and amyloidogenicity in cell culture and *in vivo* .

## Materials and Methods

### Reagents

Epigallocatechin gallate (EGCG) was purchased from Cayman Chemicals (Ann Arbor, MI, USA). CLR01 was prepared and purified as described previously [13,14].

### Recombinant TTR

Recombinant TTR Y78F was produced in a bacterial expression system and purified as described previously [15].

### *In Vitro* Studies of the Effect of CLR01 on Formation of TTR Aggregates in Cell Culture Medium

#### Detection of TTR Aggregation in Cell Culture Medium by a Dot-blot Filter Assay

Experiments were performed as described previously [16]. In brief, rat Schwannoma cells (RN22, American Type Cell Collection) stably transfected with TTR L55P cDNA were grown until 80 % confluence in the absence or presence of 1 μM CLR01 or EGCG (protein:CLR01, approximately 1:60) in the cell culture medium for ∼5 days. Then, cells were incubated for an additional 24 h, still in the presence of the compounds, but in serum-free media. TTR in the medium was quantified by enzyme-linked immunosorbent assay (ELISA), and medium aliquots corresponding to equal amounts of TTR (500 ng) were blotted onto a 0.2-μm pore cellulose acetate membrane. TTR aggregates retained on the membrane were immunodetected using rabbit anti-human TTR antibody (Dako, Glostrup, Denmark; 1:500) followed by anti-rabbit horseradish peroxidase antibody (1:1500) and enhanced chemiluminescence visualization (GE Healthcare, Buckinghamshire, UK). Dot-blots were quantified using the Bio-Rad ChemiDoc XRS system with Image Lab software (Bio-Rad, Hercules, CA, USA). Experiments were repeated at least 3 times and samples were analyzed in triplicate. All values are expressed as mean ± SD.

#### Evaluation of Cell Toxicity Induced by TTR Assemblies and its Inhibition

Rat Schwannoma cells (RN22) were propagated and maintained as described previously [16]. Briefly, 80 % confluent cells in Dulbecco’s minimal essential medium supplemented with 1 % fetal bovine serum were exposed, for 24 h, to 2 μM of TTR Y78F oligomers alone (control) or oligomers pretreated with EGCG (20 μM) or CLR01 (0.2–200.0 μM) at 37 °C for 6 days. EGCG or CLR01 in the absence of TTR Y78F were used to test whether the compounds were toxic at the concentrations used. After the treatment, cells were trypsinized and lysed using ice-cold lysis buffer containing 5 mM ethylenediaminetetraacetic acid, 2 mM ethylene glycol tetraacetic acid, 20 mM 3-(N-morpholino) propanesulfonic acid , 1 % Triton X-100, 1 mM phenylmethanesulfonyl fluoride, and a protease inhibitor mix (GE Healthcare). Cell lysates were used for determination of caspase-3 activation using the CaspACE fluorimetric 96-well plate assay system (Sigma-Aldrich, St. Louis, MO, USA). Protein concentration in lysates was determined using a Bio-Rad protein assay kit. Results are presented as normalized density ± SD.

### *In Vivo* Studies Using a FAP Mouse Model

#### Ethics Statement

All the experiments described herein were approved by the Portuguese General Veterinarian Board (authorization number 024976 from DGV-Portugal) and are in compliance with national rules and the European Communities Council Directive (86/609/EEC) for the care and handling of laboratory animals.

#### Transgenic Mice

Mice expressing human TTR V30M on a TTR-null background and heterozygous for the heat shock transcription factor 1 (HSF1), labeled hTTR V30M/HSF [17], were used for all *in vivo* experiments. Two groups of 4-month-old animals were used: animals treated with CLR01 (*n* = 14) or with vehicle (saline, *n* = 12) administered using subcutaneous osmotic minipumps (model 1004; Alzet, Cupertino, CA, USA). Following anesthesia with ketamine/medetomidine, the pumps were surgically implanted on the dorsal back of mice. The compound was delivered at a dose of 1.2 mg/kg/day for 35 days. After the treatment, mice were sacrificed and tissues, specifically, whole gastrointestinal (GI) tract, including stomach, colon, and duodenum, and dorsal root ganglia (DRG) were immediately excised and frozen at –70 º C, or fixed in 4 % neutral buffered formalin and embedded in paraffin for light microscopy analysis.

#### Determination of TTR Concentration Levels in Mouse Plasma by ELISA

TTR plasma concentrations were determined by ELISA, as described previously [18]. Briefly, 96-well plates were coated overnight at 4 °C with rabbit anti-human TTR polyclonal antibody (Abcam, Cambridge, UK). After blocking and washes, TTR standards (2–25 ng/ml) and diluted mouse plasma samples were applied in triplicate and incubated for 2 h at room temperature. Next, sheep anti-human TTR polyclonal antibody (Abcam) was added and incubated for 1 h. After washing, the plate was incubated with anti-sheep antibody-conjugated alkaline phosphatase. p-Nitrophenyl phosphate was employed for color development. The absorbance was measured at 405 nm and data were fitted to a second-order polynomial (quadratic equation).

#### Analysis of Competition of CLR01 with T_4_ for the Binding to Plasma TTR by Native Gel Electrophoresis

Five-microliter aliquots of plasma from mice treated with vehicle or CLR01 were incubated with [^125^I]-T_4_ (specific radioactivity 1250 μCi/μg; Perkin-Elmer, Waltham, MA, USA). Then, plasma proteins were fractionated by native polyacrylamide gel electrophoresis (PAGE), as described previously [19]. The gel was dried, subjected to phosphor imaging (Typhoon 8600; Molecular Diagnostics, Amersham Biosciences, Uppsala, Sweden), and the intensity of the bands determined using ImageQuant v. 5.1.

#### Isoelectric Focusing Under Partially Dissociating Conditions

Twenty-five microliters of plasma from CLR01- and vehicle-treated mice were subjected to native PAGE. The TTR gel band in each lane was excised and used for a pH 4.0–6.5 isoelectric focusing (IEF) polyacrylamide gel run for 6 h at 1200 V under partially dissociating conditions (4 M urea) [19]. Proteins were stained with Coomassie Blue. The gels were scanned and analyzed by densitometry using ImageQuant v. 5.1.

#### Immunohistochemistry

Five-mm-thick tissue sections were deparaffinated in Histoclear and hydrated in a descending alcohol concentration series. Endogenous peroxidase activity was quenched with 3 % hydrogen peroxide in methanol, and sections were blocked in 4 % fetal bovine serum and 1 % bovine serum albumin in phosphate-buffered saline. The primary antibodies and the respective dilutions used were as follows: rabbit polyclonal anti-TTR (1:1000) (Dako); goat polyclonal anti-binding immunoglobulin protein (BiP) (1:50) and rabbit polyclonal anti-Fas (1:200) (Santa Cruz Biotechnology, Santa Cruz, CA, USA); rabbit polyclonal anti-3-nitrotyrosine (1:500) (Chemicon, Temecula, CA, USA). Antibodies were diluted in blocking solution and incubated overnight at 4 °C. Slides were subsequently incubated with a biotin–extravidin enzyme complex (ABC Elite Vectastain kit; Vector Laboratories, Burlingame, CA, USA) using hydrogen peroxide and diaminobenzidine as substrate and chromogen, respectively. Immunohistochemistry (IHC) analysis was carried out independently by two investigators blinded to the origin of the tested tissue sections. Semi-quantitative immunohistochemical analysis was performed using Image-Pro Plus v. 5.1. Each slide was analyzed in 5 different representative areas.

#### Western Blot Analysis

Disease-relevant tissues, including DRG, stomach, and colon, were homogenized on ice using a small glass rod homogenizer in lysis buffer (as in the section ‘Evaluation of Cell Toxicity Induced by TTR Assemblies and its Inhibition’). After centrifugation at 18,700 g for 20 mins at 4 °C, protein concentration in the supernatant was determined by the Bradford protein assay (Bio-Rad). Fifty micrograms of total protein from each tissue sample was fractionated on 15 % sodium dodecyl sulfate-PAGE and transferred onto nitrocellulose Hybond-C membranes using the Mini Trans-Blot Cell (Bio-Rad) system. The primary antibodies and the respective dilutions used were as follows: rabbit polyclonal anti-TTR (1:1000; Dako, Carpinteria, CA, USA); rabbit polyclonal anti-BiP (1:1000) (Abcam); mouse monoclonal anti-glyceraldehyde 3-phosphate dehydrogenase (GAPDH) (1:1000) (Abcam). Blots were visualized using enhanced chemiluminescence (Amersham ECL Prime) and quantified as described in ‘Detection of TTR Aggregation in Cell Culture Medium by a Dot-blot Filter Assay’. Immunosignals were normalized to GAPDH expression. Each group was compared with control. Results are presented as normalized density ± SD. A *p*-value < 0.05 was considered statistically significant.

## Results

### CLR01 Inhibits TTR Aggregation in a Cell Culture System

**Fig. 1:**
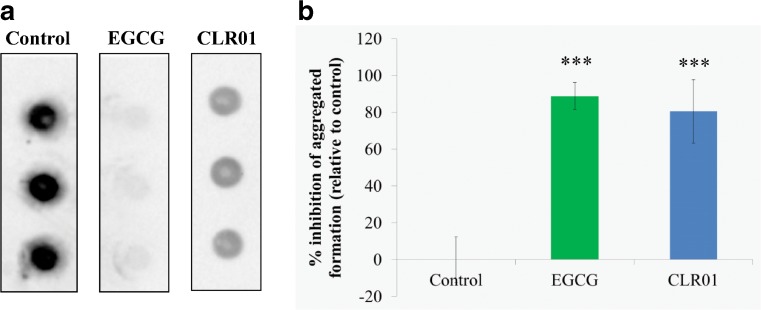
CLR01 inhibits transthyretin (TTR) aggregation in a cell culture system. (**A**) Immunodetection of TTR oligomers in a dot-blot filtration assay of conditioned medium from TTR L55P-transfected Schwannoma RN22 cells grown in the absence (control) or in the presence of CLR01 or epigallocatechin gallate (EGCG). (**B**) Densitometric analysis of the dot-blot assay showing the percentage of inhibition of aggregate formation by each compound, compared with the control (0 % inhibition) (***p < 0.005)

The effect of CLR01 on TTR aggregation was investigated in a rat Schwannoma cell line (RN22) transfected with TTR L55P, which secretes the TTR variant to the medium where it aggregates [16]. The cells were grown in the absence or presence of CLR01 or of EGCG, which has previously been found to be a potent inhibitor of TTR aggregation [20,21]. Then, cell culture media aliquots were spotted on a cellulose–acetate membrane, which retains TTR aggregates, but not soluble TTR. The membranes were probed with an anti-TTR antibody. The results are shown in Fig. 1.

In agreement with previous studies [20,21] dot-blot of conditioned medium obtained in the presence of EGCG revealed almost complete inhibition of TTR aggregation (88.74 ± 7.41 %, *p* < 0.001). Similarly, conditioned medium from cells incubated in the presence of CLR01 presented significantly fewer aggregates than media from control cells (80.50 % ± 17.10, *p* < 0.001).

### CLR01 Protects Neuronal Cells Against TTR-induced Toxicity

To investigate the impact of CLR01 on the toxicity of TTR assemblies we performed a standardized caspase-3 assay in RN22 cells exposed to TTR oligomers formed in the absence or presence of CLR01, or EGCG as a positive control. Cells were exposed to TTR Y78F oligomers pretreated for 24 h with EGCG or CLR01, or with TTR incubated in the absence of inhibitors as a negative control. Different concentrations of CLR01 were tested, and the toxicity of CLR01 and EGCG at the same concentration in the absence of TTR was also assayed.

**Fig. 2:**
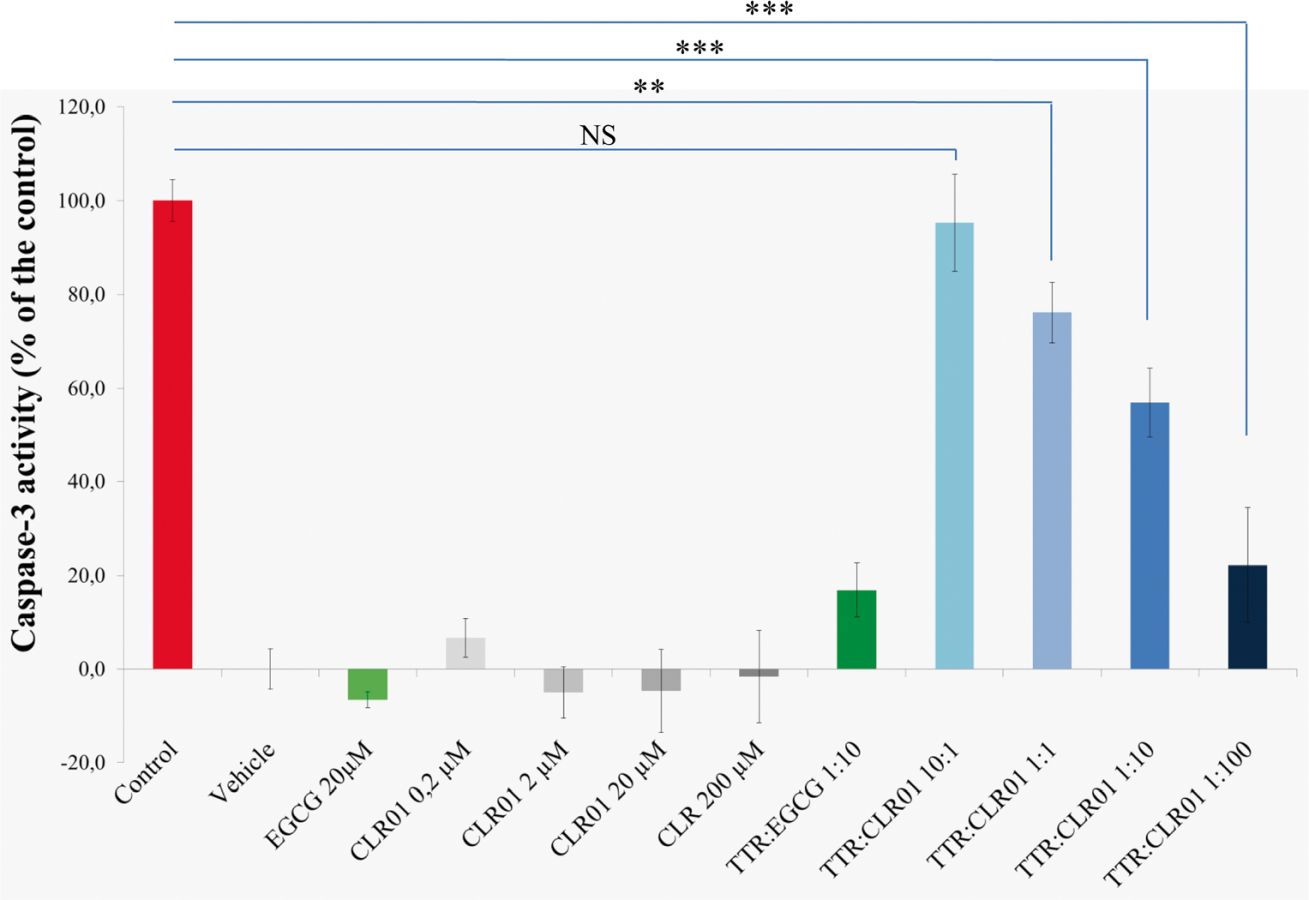
CLR01 inhibits transthyretin (TTR)-induced toxicity in a-dose dependent manner. Activation of caspase-3 in Schwannoma RN22 cells exposed to 2 μM TTR Y78F oligomers or TTR Y78F oligomers pretreated with epigallocatechin gallate (EGCG) (20 μM) or CLR01 (0.2–200.0 μM) for 24 h. In parallel, cells were incubated with EGCG or CLR01 alone, at the same concentrations (**p < 0.01; ***p < 0.005). NS = nonsignificant

As expected [21], pretreatment of TTR Y78F oligomers with EGCG resulted in strong inhibition of caspase-3 activation (16.86 % ± 5.75 % of the control activity) (Fig. 2). Pre-incubation of TTR Y78F with CLR01 also protected the cells against the toxicity of the TTR oligomers in a concentration-dependent manner, decreasing caspase-3 activity to a minimum of 22.17 % ± 12.20 relative to control at 200 μM (TTR: CLR01 concentration ratio 1:100; Fig. 2). Thus, CLR01 was found to be about 1 order of magnitude less potent inhibitor of TTR oligomer-induced cytotoxicity when compared with EGCG in this particular cell culture system.

### Evaluation of CLR01 Effect in a Mouse Model of FAP

In view of the promising *in vitro* results described above, we investigated the effect of peripherally administered CLR01 on the deposition of TTR and associated biomarkers using a FAP mouse model expressing the amyloidogenic human TTR V30M variant on a HSF1-null background [17]. The lack of HSF1 expression leads to an extensive and early deposition of nonfibrillar TTR in different organs, including the GI tract and the PNS. TTR aggregates start to deposit at 3 months of age and evolve to fibrillar, congophilic material typically by 1 year of age. Therefore, this mouse model is highly relevant to testing new therapeutic strategies targeting different stages of the disease. In this study, we aimed at evaluating the effect of CLR01 at a very early stage of the disease in which deposition of nonfibrillar TTR occurs in different tissues.

Four-month-old hTTR V30M/HSF mice were treated for 35 days with CLR01 (1.2 mg/kg/day) in saline as vehicle (*n* = 14) or vehicle alone (*n* = 12) using subcutaneously implanted osmotic minipumps. Protocol design, drug dosage, and selection of endpoints were based on previous studies [22,23]. The CLR01 dosage used in this study was not expected to cause adverse side effects based on previous studies [23]. Indeed, no difference was observed in body weight or mortality between animals treated with CLR01 and age-matched vehicle-treated controls (Supplementary Table 1). In addition, no histological abnormalities were observed in liver sections stained with hematoxylin and eosin for morphologic assessment.

### CLR01 Does not Interfere With the Transport of T_4_ by the Thyroid Hormone Serum Transport Proteins

To assess if CLR01 interacts with TTR at the T_4_ binding site *in vivo*, plasma from mice treated with CLR01 or vehicle was incubated with radiolabeled T_4_ (^125^I-T_4_) and subjected to gel electrophoresis under native conditions. T_4_-binding proteins were visualized by phosphor imaging analysis, as shown in Supplementary Fig. S1.A. Two main T_4_ binding proteins were detected corresponding to albumin (the main mouse plasma T_4_ binding protein) and TTR. Binding of T_4_ to T_4_-binding globulin in mouse plasma was only residual. The results showed that the relative intensity of the protein bands from treated and untreated mice was similar in both cases (Supplementary Fig. S1.B), indicating that CLR01 did not bind to TTR at the T_4_ binding sites.

### CLR01 Does not Impact the Native TTR Tetramer Stability

IEF, under partially dissociating conditions (4 M urea), allows visualization of different plasma TTR species, including monomer, an oxidized monomer, and several lower-pI bands corresponding to tetramers. Stabilization of the native TTR conformation by small molecules has been associated with higher tetramer/total protein ratios [19]. Densitometry analysis of the IEF gels demonstrated that CLR01 treatment did not increase plasma TTR resistance to dissociation when compared with plasma TTR from control animals under the tested conditions (Supplementary Fig. S2.A, B).

### CLR01 Reduces Extracellular TTR Deposition and Rescues Tissue Damage Without Adverse Effects

TTR levels in plasma from vehicle-treated and CLR01-treated mice were determined by ELISA and showed no statistical difference between the two groups (504 ± 123 μg TTR/ml and 560 ± 136 μg TTR/ml, respectively), suggesting that CLR01 treatment did not affect TTR turnover *in vivo*.

To address the efficacy of CLR01 in preventing TTR deposition and tissue damage, several tissues were analyzed by IHC or Western blot to evaluate the levels of TTR burden and associated biomarkers. At the end of the treatment, the mice were ∼5.5 months old. As expected at this age, vehicle-treated mice presented extensive TTR staining throughout the interstitial connective tissue of several organs, with particular involvement of the PNS. In the DRG, TTR aggregates were found extracellularly surrounding the perikaryon in close contact with the satellite glial cells. These observations were in agreement with previous reports [17], and simulate the pathological findings of FAP.

**Fig. 3:**
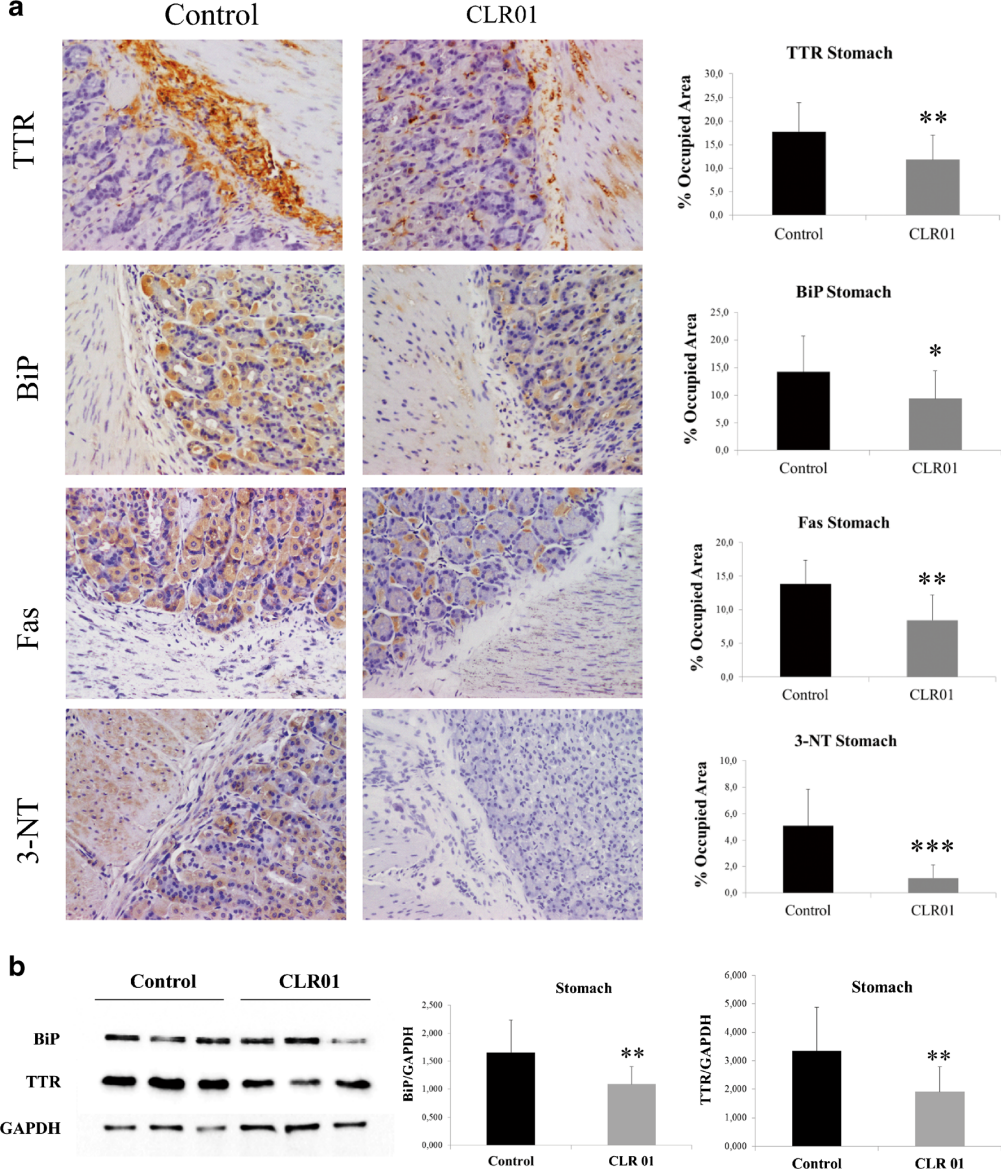
CLR01 decreases transthyretin (TTR) burden and associated toxicity in the stomach of heat-shock transcription factor (HSF)-1–TTR V30M/HSF mice. (**A**) Representative immunohistochemistry analysis of TTR, binding immunoglobulin protein (BiP), Fas, and 3-nitrotyrosine in stomach of mice treated with CLR01 (right panels; *n* = 14) and age-matched controls (left panels; *n* = 12); 20× magnification. Bar graphs: quantification of immunohistochemical images is represented as percentage of occupied area ± SD (**p < 0.01; ***p < 0.005). (**B**) Representative anti-BiP and anti-TTR Western blots of stomachs from CLR01- and vehicle-treated mice. Bar graphs: normalized BiP/glyceraldehyde 3-phosphate dehydrogenase (GAPDH) and TTR/GAPDH density quantifications ± SD (*p < 0.05; **p < 0.01)

**Fig. 4:**
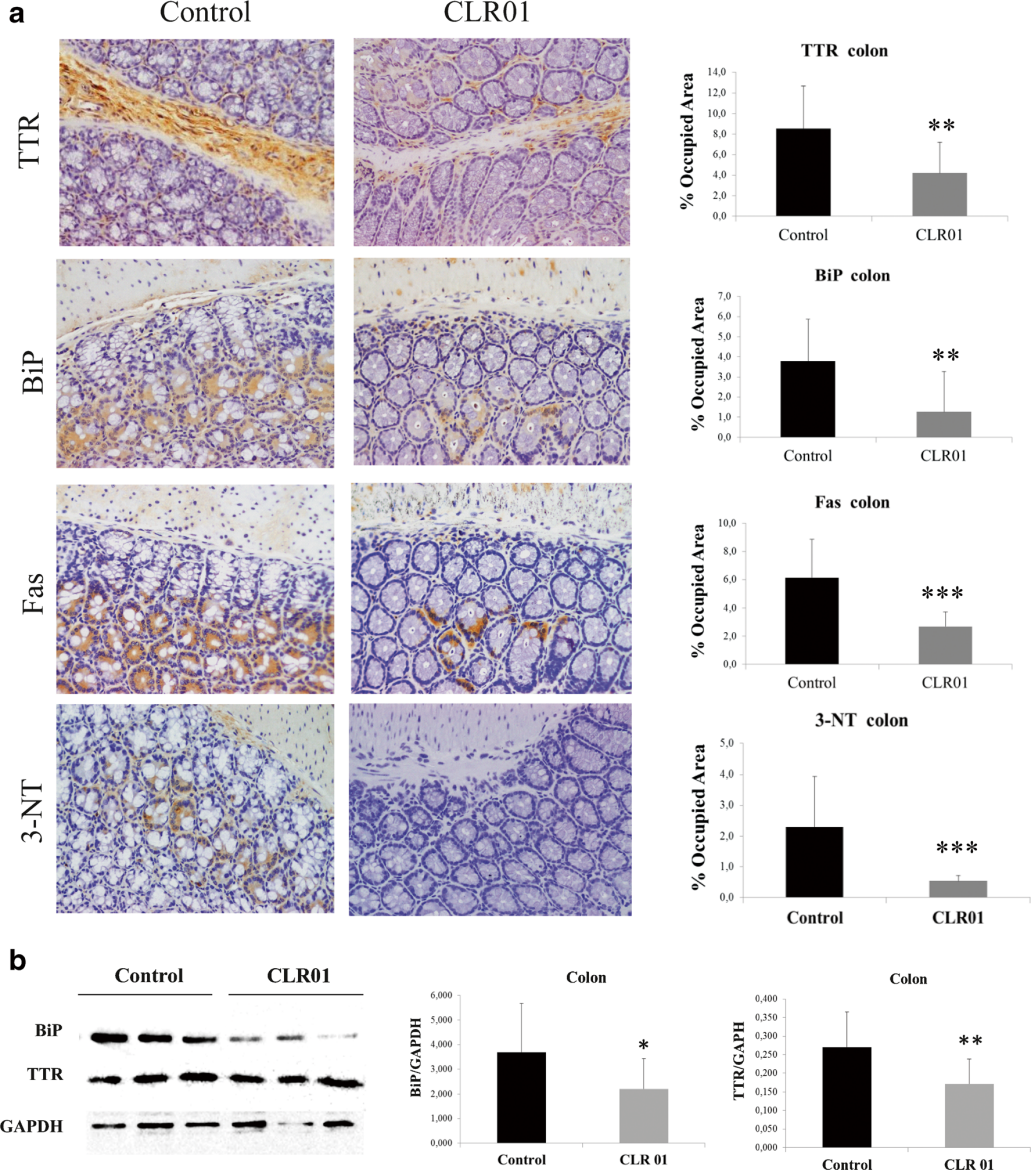
CLR01 decreases transthyretin (TTR) burden and associated toxicity in colon of hTTR V30M/HSF mice. (**A**) Illustrative immunohistochemistry analysis of TTR, binding immunoglobulin protein (BiP), Fas, and 3-nitrotyrosine in colon of mice treated with CLR01 (right panels; *n* = 14) and age-matched controls (left panels; *n* = 12); 20× magnification. Bar graph: quantification of immunohistochemical images is presented as percentage of occupied area ± SD (**p < 0.01; ***p < 0.005). (**B**) Representative anti-BiP and anti-TTR Western blots of colon from CLR01- and vehicle-treated mice. Bar graph: normalized BiP/GAPDH and TTR/GAPDH density quantifications ± SD (*p < 0.05; **p < 0.01)

**Fig. 5:**
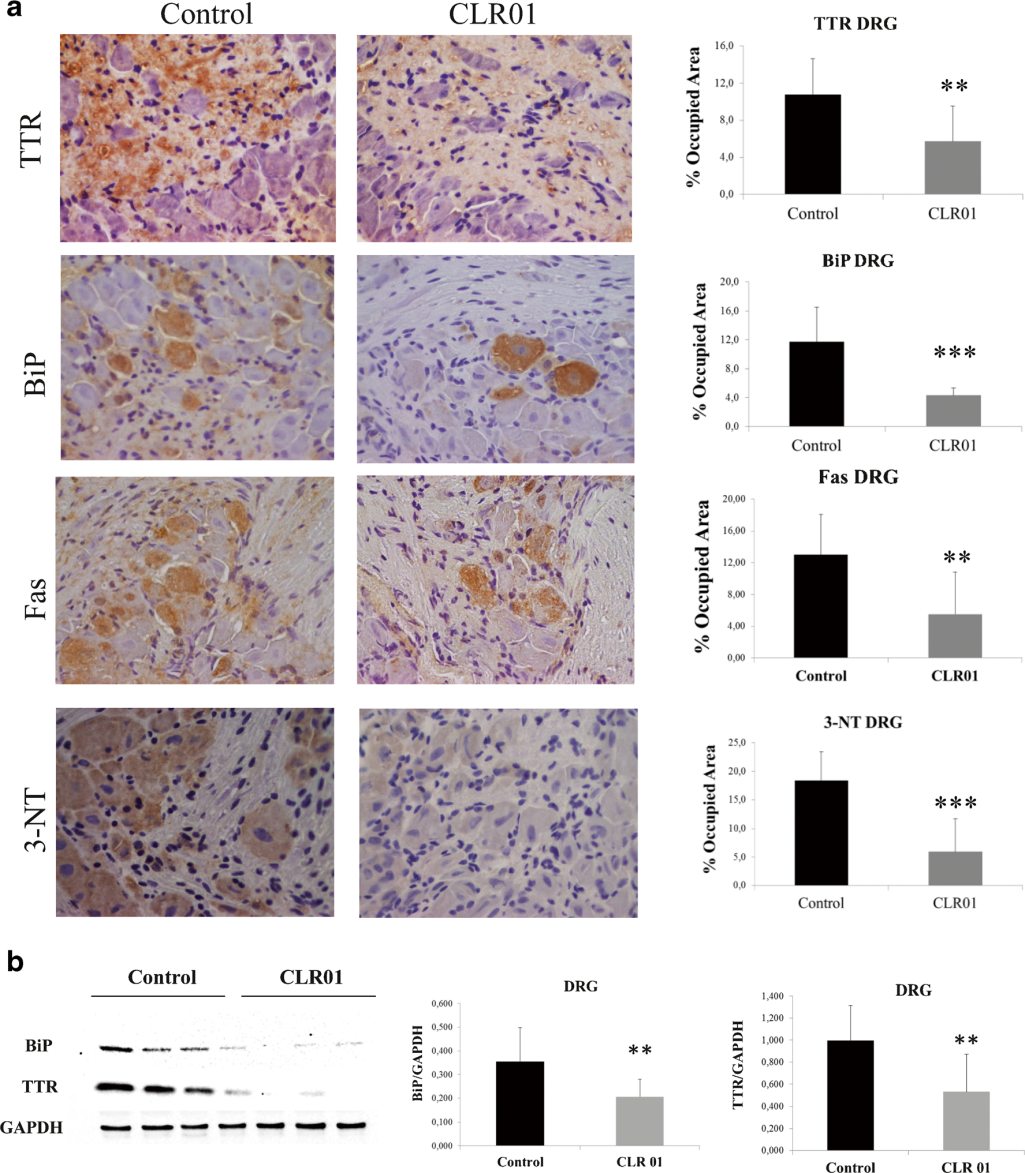
CLR01 decreases transthyretin (TTR) burden and associated toxicity in dorsal root ganglia (DRG) of hTTR V30M/HSF mice. (**A**) Representative immunohistochemistry analysis of TTR, binding immunoglobulin protein (BiP), Fas, and 3-nitrotyrosine in DRG of mice treated with CLR01 (right panels; *n* = 14) and age-matched controls (left panels; *n* = 12); 20× magnification. Bar graph: quantification of immunohistochemical images is presented as percentage of occupied area ± SD (**p < 0.01; ***p < 0.005). (**B**) Representative anti-BiP and anti-TTR Western blots of DRG from CLR01- and vehicle-treated mice. Bar graph: normalized BiP/GAPDH and TTR/GAPDH density quantifications ± SD (**p < 0.01)

In contrast, after 5 weeks of subcutaneous administration of CLR01, hTTR V30M/HSF mice showed a significant reduction of TTR deposition in all target organs analyzed by IHC: 33 % in stomach (% occupied area: controls 17.79 % ± 6.18 % *vs* CLR01-treated 11.84 % ± 5.27 %, *p* < 0.01; Fig. 3A), 51 % in colon (controls 8.53 % ± 4.13 %, CLR01-treated 4.22 ± 3.00, *p* < 0.01; Fig. 4A), and 47 % in DRG (controls 10.76 % ± 3.87 %, CLR01-treated 5.74 % ± 3.81 %, *p* < 0.01; Fig. 5A). These results were further supported by Western blot analysis of TTR in whole-tissue extracts (Figs 3B, 4B, and 5B, respectively).

Accumulation of amyloidogenic proteins, such as TTR, in the extracellular space leads to membrane permeabilization. Consequently, perturbation of intracellular calcium homeostasis has been proposed as a common primary pathogenic event responsible for initiating several pathogenic signaling pathways in many amyloid-related disorders [24]. To investigate if inhibition of extracellular deposition of TTR in CLR01-treated mice reduced TTR-induced tissue injury, we analyzed tissues for activation of several disease markers, including endoplasmic reticulum–BiP, the Fas/CD95 death receptor, and 3-nitrotyrosine [25–27].

Compared with vehicle-treated mice, we found a 34 % reduction in the BiP-stained area of the stomach of CLR01-treated mice (% occupied area: controls 14.25 % ± 6.46 % *vs* CLR01-treated 9.43 ± 5.05, *p* < 0.05; Fig. 3A) and an even higher decrease in colon (67 %; controls 3.78 ± 2.10, CLR01-treated 1.26 ± 2.00, *p* < 0.01; Fig. 4A) and DRG (63 %; controls 11.69 % ± 4.85 %, CLR01-treated 4.31 % ± 1.05 %, *p* < 0.001; Fig. 5A). Western blot evaluation of BiP levels revealed a similar trend, as shown in Figs 3B, 4B and 5B, respectively.

We next evaluated Fas/CD95 death receptor immunostaining and found a significant decrease in Fas-mediated apoptosis in CLR01-treated mice compared with the vehicle-treated group in stomach (39 % reduction, *p* < 0.001; Fig. 3A), colon (56 % reduction, *p* < 0.001; Fig. 4A), and DRG (68 % reduction, *p* < 0.001; Fig. 5A).

TTR-induced cytotoxicity also was addressed by analysis of 3-nitrotyrosine, one of the most common products of tyrosine nitration mediated by reactive nitrogen species. The results showed a strong decrease in nitric oxide-dependent oxidative stress throughout the GI tract in CLR01-treated mice relative to vehicle-treated mice [stomach: 78 % reduction, *p* < 0.001 (Fig. 3A); colon: 76 % reduction, *p* < 0.001 (Fig. 4A] and DRG (68 % reduction, *p* < 0.001; Fig. 5A).

## Discussion

Over the last few years, many small compounds have been proposed as disease-modifying therapeutic agents specific for TTR-related amyloidosis. Among those, Tafamidis, a kinetic stabilizer of TTR dissociation, has completed phase II/III trials for the treatment of FAP.

However, different compounds may be needed for effective stabilization of different TTR mutants and, in this sense, more recently, several nonspecific amyloid inhibitors have been reported, as is the case of EGCG [20–22]. Similarly, CLR01 was found to be an efficient inhibitor of aggregation of 9 different amyloidogenic proteins, *in vitro*, one of which was TTR [12].

Here, we extended the examination of CLR01 as a potential disease-modifying agent for TTR-related amyloidosis, first by comparing its effect side-by-side with EGCG *in vitro*. The two compounds were tested for their capability in modulating TTR abnormal folding and toxicity in neuronal culture systems. We found that CLR01 strongly suppressed TTR aggregation and alleviated cultured cells from the neurotoxic effect induced by extracellular oligomeric TTR in a dose-dependent manner. Because CLR01 has been shown to bind to amyloidogenic proteins already at the monomer stage [12], we hypothesize that it remodels early, partially unfolded TTR monomers and/or oligomers into nontoxic species and prevents their aggregation. In addition, CLR01 may interfere with the interaction of TTR assemblies with membrane targets.

In both the inhibition of self-assembly (Fig. 1) and inhibition of toxicity (Fig. 2) assays, EGCG was more effective than CLR01, though this effect was substantially more pronounced in the caspase-inhibition assay. A likely explanation is the longer incubation time used in that assay relative to the aggregation inhibition assay. Recently, Palhano et al. [28] showed that EGCG binds covalently to the target protein, and this reaction reaches completion within several hours. Therefore, by the time the protein–inhibitor mixture was added to the cell culture, essentially all of the TTR was already covalently modified by EGCG. In contrast, CLR01 binds noncovalently and its binding is highly labile [29]. Thus, the different nature of the interaction of the 2 compounds with TTR might be responsible for the difference of inhibition efficiency under the conditions of the assay as 10-times higher concentrations of CLR01 were required to achieve the same level of inhibition found for EGCG (Fig. 2). Similarly to the results observed here, Sinha et al. [30] found that CLR01 and EGCG had an analogous inhibitory effect on Aβ self-assembly, yet EGCG was more effective than CLR01 in preventing Aβ-induced toxicity in several culture systems, likely owing to the differences in mechanism of action described above. In addition, it is possible that the ability of EGCG to modulate multiple signaling pathways associated with neurodegeneration [31,32] and to scavenge free radicals [32] provided additional neuroprotective activities, which are not shared by CLR01.

**Fig. 6:**
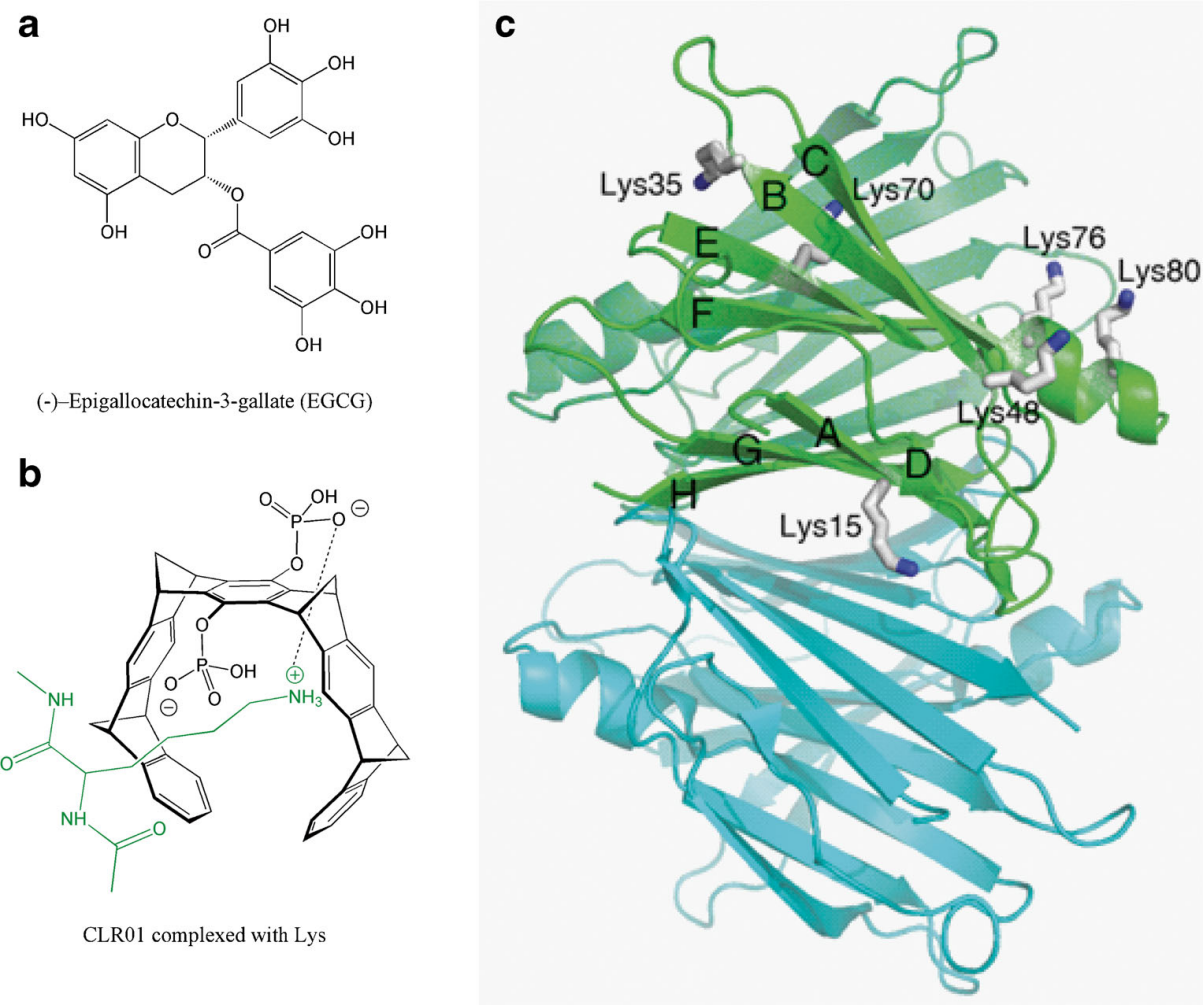
(**A**) Chemical structure of epigallocatechin gallate (EGCG) and (**B**) CLR01 complexed with lysine. (**C**) Crystal structure of transthyretin [PDB 2H4E] highlighting the lysine residues in one of the monomers. Each monomer has a β-sandwich structure composed of two β-sheets (DAGH and CBEF) and a short helix. Lys9 and Lys126 are located in the disordered N- and C-terminus (not shown)

Considering the chemical structure of CLR01 and EGCG, they share little structural similarities and apparently act through distinct mechanisms. CLR01 binds specifically to exposed lysine residues with μM affinity and with lower affinity to arginines (Fig. 6A, B). In contrast, EGCG has been reported to interact nonspecifically with amyloidogenic proteins through hydrophobic interactions and hydrogen bonding, and to not bind monomers to a significant extent [33,34], likely because its binding sites are grooves in the β-sheet structure that are characteristic of amyloids and “fibrillar oligomers”. However, recently, EGCG was also reported to bind covalently to cysteinyl thiol residues, and to free amines of the N-terminus or of lysine side chains through auto-oxidation [28]. Thus, even though both compounds can bind to lysine residues, CLR01 binds reversibly, regardless of assembly state, whereas EGCG binds to larger assemblies and attaches to them irreversibly.

In view of the *in vitro* results described above and recent studies showing a therapeutic effect for CLR01 in animal models of Alzheimer’s disease (AD) [23] and Parkinson’s disease [35], we examined the impact of CLR01 on pathogenic TTR deposition in a mouse model of FAP [17] at early stages of disease.

In this study, we assessed the effect of CLR01 on TTR deposition throughout the GI tract and the PNS, and found a significant decrease of TTR burden in all the organs analyzed, particularly in the intestine, stomach, and DRG. These results were further supported by a substantial decrease of TTR deposition-associated endoplasmic reticulum stress response, apoptosis, and protein oxidation, indicating that reduction of TTR load by CLR01 correlates with decreased proteotoxicity. In FAP, neuropathy begins at the earliest stages of the disease when nonfibrillar TTR deposits in the PNS. Analysis of nerve biopsy samples from asymptomatic FAP patients showed upregulation of pro-inflammatory cytokines, extracellular matrix remodeling mediators, and inducible nitric oxide synthase [25,36]. These pathogenic responses to toxic TTR species are similar to those observed in other neurodegenerative diseases. For example, oligomeric β-amyloid (Aβ) induces a potent inflammatory response, which suppresses microglial phagocytosis and uptake of Aβ fibrils, thereby contributing to early neurodegeneration of AD pathology [37]. Our data clearly indicate that CLR01 rescues FAP mice from neuronal injury caused by abnormal protein deposition, similarly to previously reported data in an AD mouse model [23]. Therefore, it seems reasonable to speculate that following CLR01 treatment, a restored extracellular environment might improve TTR clearance by the innate immune system. Supporting this speculation, in a zebrafish model expressing human, WT α-synuclein, inhibition of the 26S ubiquitin–proteasome system by oligomeric or aggregated α-synuclein was alleviated by treatment with CLR01, leading to a marked reversal of α-synuclein accumulation [35].

Though we did not compare the effects of CLR01 and EGCG side-by-side *in vivo,* it is interesting to compare the data obtained here to a previous study of EGCG in hTTR V30M/HSF mice of the same age [22]. Direct comparison is not possible because the 2 compounds were administered through different routes and at different doses [22,23]. Neither compound affected TTR plasma concentration levels, but EGCG appeared to increase TTR tetramer levels, whereas CLR01 did not. On average, the effect of EGCG on TTR deposition and on the levels of the associated biomarkers, BiP, Fas/CD95, and 3-nitrotyrosine, was moderately larger than that of CLR01. At the doses administered, EGCG and CLR01 are expected to reach plasma steady-state levels of 280 nM [38] and 170 nM [23], respectively. Thus, given the lower dose and shorter treatment period of CLR01, we conclude that the impact of the two compounds on FAP pathology in this mouse model is similar, despite their different mechanisms of action.

Lysine residues are known to play a key role in the aberrant self-association of many amyloidogenic proteins and have been involved in anchoring/insertion of amyloid intermediaries to membranes through electrostatic interaction with the negatively charged phosphate groups of phospholipids [39–41]. Therefore, exposed lysine residues have been proposed as attractive molecular targets for the development of new drugs for the prevention/treatment of amyloidosis [12, 42].

TTR has 8 lysine residues in each of the 4 monomers comprising the active tetramer (Fig. 6C). All of them are exposed to the solvent, either on the external surfaces of the β-sandwich arrangement of the monomer (Lys 15 at the DAGH sheet, and Lys 35, 48, and 70 at the CBEF sheet) or positioned at the exposed α-helix (Lys 76 and Lys 80) and N- and C-terminus (Lys 9, Lys 126). Thus, in principle, all lysine residues can bind to compounds such as CLR01, and it is conceivable that binding to the Lys located in the penultimate β-strands (especially Lys 15) would inhibit end-to-end alignment of the non-native monomers and interfere with lateral alignment of protofilaments in the tight assembly characteristic of amyloid formation. Precise determination of the binding sites(s) of CLR01 on TTR will require additional investigation.

Despite many recent advances, prevention of, or treatment for, TTR-related amyloidosis is highly complex and likely will require diverse approaches. Combination therapies acting simultaneously on different molecular targets might increase treatment effectiveness and improve clinical outcome. This study demonstrates that CLR01 modulates early stages of TTR self-assembly and inhibits TTR-induced neurotoxicity. Our preclinical data support previous studies showing the beneficial effects of CLR01 administration in AD and Parkinson’s disease-like pathology *in vivo*, and highlight CLR01 as a broad, process-specific inhibitor of amyloid formation. Compound formulation is expected to improve its pharmacological properties towards potential clinical usefulness.

## Electronic supplementary material

Below is the link to the electronic supplementary material.

ESM 1 (PDF 2095 kb)

Supplementary Table 1 Treatment of CLR01 does not impact body weight (% of pre-operative baseline). Body weight of CLR01- or vehicle-treated hTTR V30M/HSF mice was monitored every week during the entire experimental period. The table shows percentage % of pre-operative baseline. NS = not significant (JPEG 16 kb)

High resolution image (TIFF 795 kb)

Fig. S1 CLR01 does not interfere with thyroxine (T4) transport by peripheral transthyretin (TTR). (A) Representative polyacrylamide gel electrophoresis analysis of [125I]-T4 distribution among T4-binding proteins after incubation with plasma from CLR01-t and vehicle-treated hTTR V30M/HSF mice. Plasma thyroid hormone transport proteins are indicated. (B) The bar graph shows percentage of total bound [125I]-T4 to each plasma T4-binding protein. N.S. = not significant; TBG = thyroxine binding globulin (JPEG 35 kb)

High resolution image (TIFF 3431 kb)

Fig. S2 CLR01 does not affect plasma transthyretin (TTR) dissociation under partially denaturing conditions. Plasma from hTTR V30M/HSF mice treated with CLR01 and vehicle were subjected to isoelectric focusing analysis (IEF) under partially denaturing conditions (4 M urea). Under these conditions different protein bands corresponding to TTR monomers, an oxidized form of the monomer, and tetramers are visualized. (B) TTR tetramer/total TTR bands ratio obtained after densitometry analysis of IEF gels. N.S. = not significant (JPEG 33 kb)

High resolution image (TIFF 4301 kb)
