# Supplementary figures and images for: Molecular Tweezers Targeting Transthyretin Amyloidosis
Source: Neurotherapeutics. 2014 Jan 24;11(2):450–61. doi: 10.1007/s13311-013-0256-8 (PMC3996111; doi:10.1007/s13311-013-0256-8)

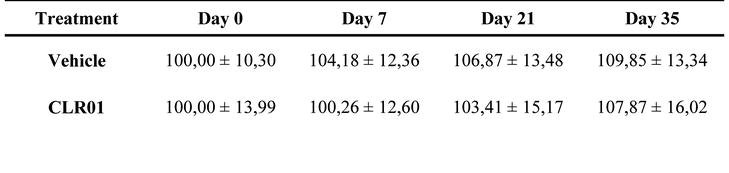

Supplement: Supplementary file 2 — Treatment of CLR01 does not impact body weight (% of pre-operative baseline). Body weight of CLR01- or vehicle-treated hTTR V30M/HSF mice was monitored every week during the entire experimental period. The table shows percentage % of pre-operative baseline. NS = not significant (JPEG 16 kb) [file 13311_2013_256_Fig7_ESM.jpg]

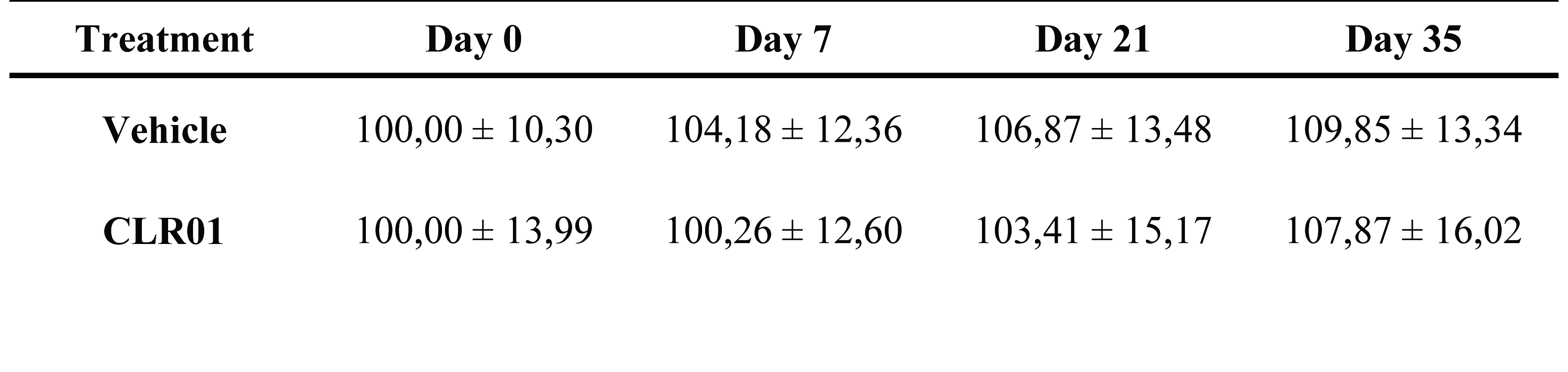

Supplement: Supplementary file 3 — High resolution image (TIFF 795 kb) [file 13311_2013_256_MOESM2_ESM.tif]

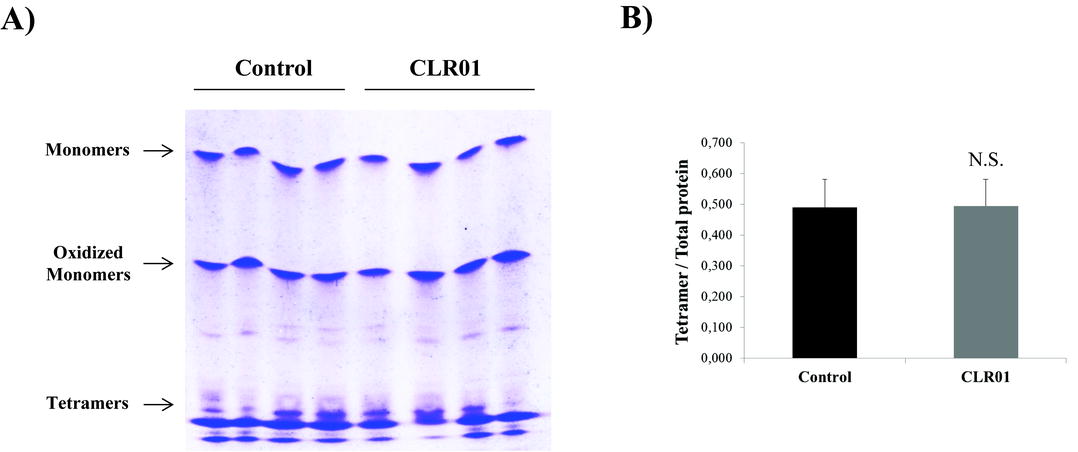

Supplement: Supplementary file 4 — CLR01 does not interfere with thyroxine (T4) transport by peripheral transthyretin (TTR). (A) Representative polyacrylamide gel electrophoresis analysis of [125I]-T4 distribution among T4-binding proteins after incubation with plasma from CLR01-t and vehicle-treated hTTR V30M/HSF mice. Plasma thyroid hormone transport proteins are indicated. (B) The bar graph shows percentage of total bound [125I]-T4 to each plasma T4-binding protein. N.S. = not significant; TBG = thyroxine binding globulin (JPEG 35 kb) [file 13311_2013_256_Fig8_ESM.jpg]

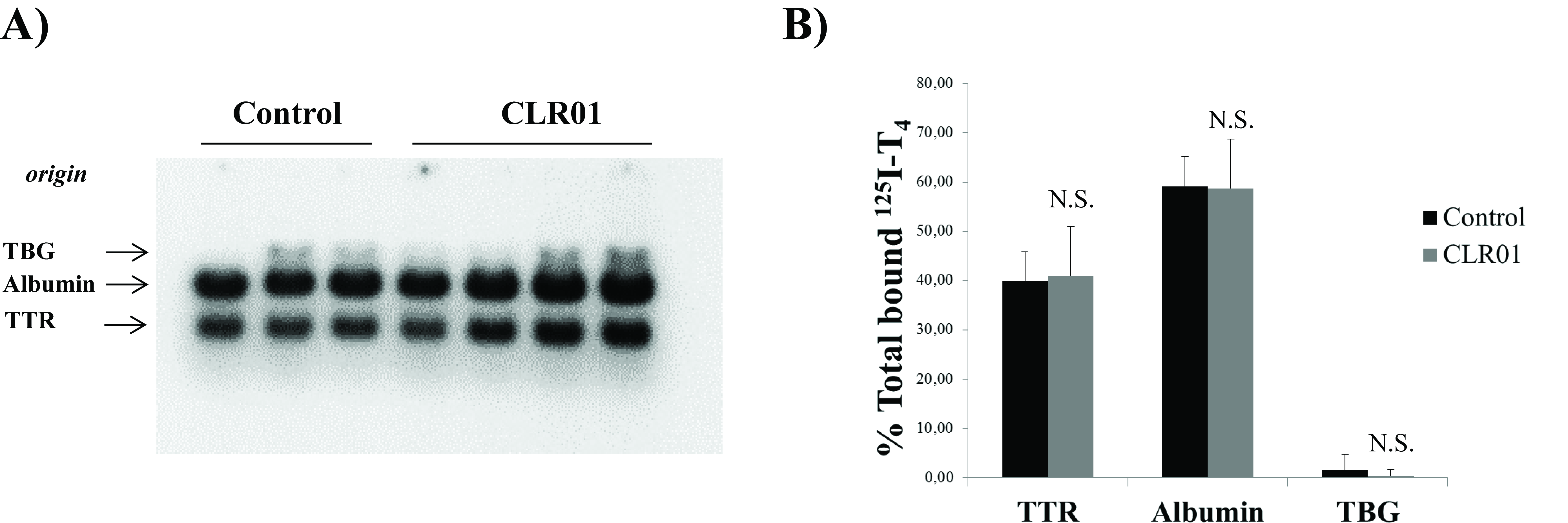

Supplement: Supplementary file 5 — High resolution image (TIFF 3431 kb) [file 13311_2013_256_MOESM3_ESM.tif]

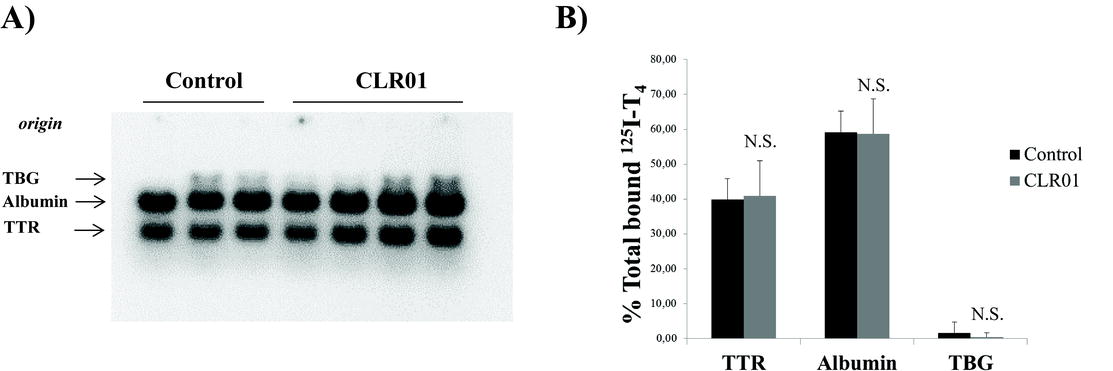

Supplement: Supplementary file 6 — CLR01 does not affect plasma transthyretin (TTR) dissociation under partially denaturing conditions. Plasma from hTTR V30M/HSF mice treated with CLR01 and vehicle were subjected to isoelectric focusing analysis (IEF) under partially denaturing conditions (4 M urea). Under these conditions different protein bands corresponding to TTR monomers, an oxidized form of the monomer, and tetramers are visualized. (B) TTR tetramer/total TTR bands ratio obtained after densitometry analysis of IEF gels. N.S. = not significant (JPEG 33 kb) [file 13311_2013_256_Fig9_ESM.jpg]

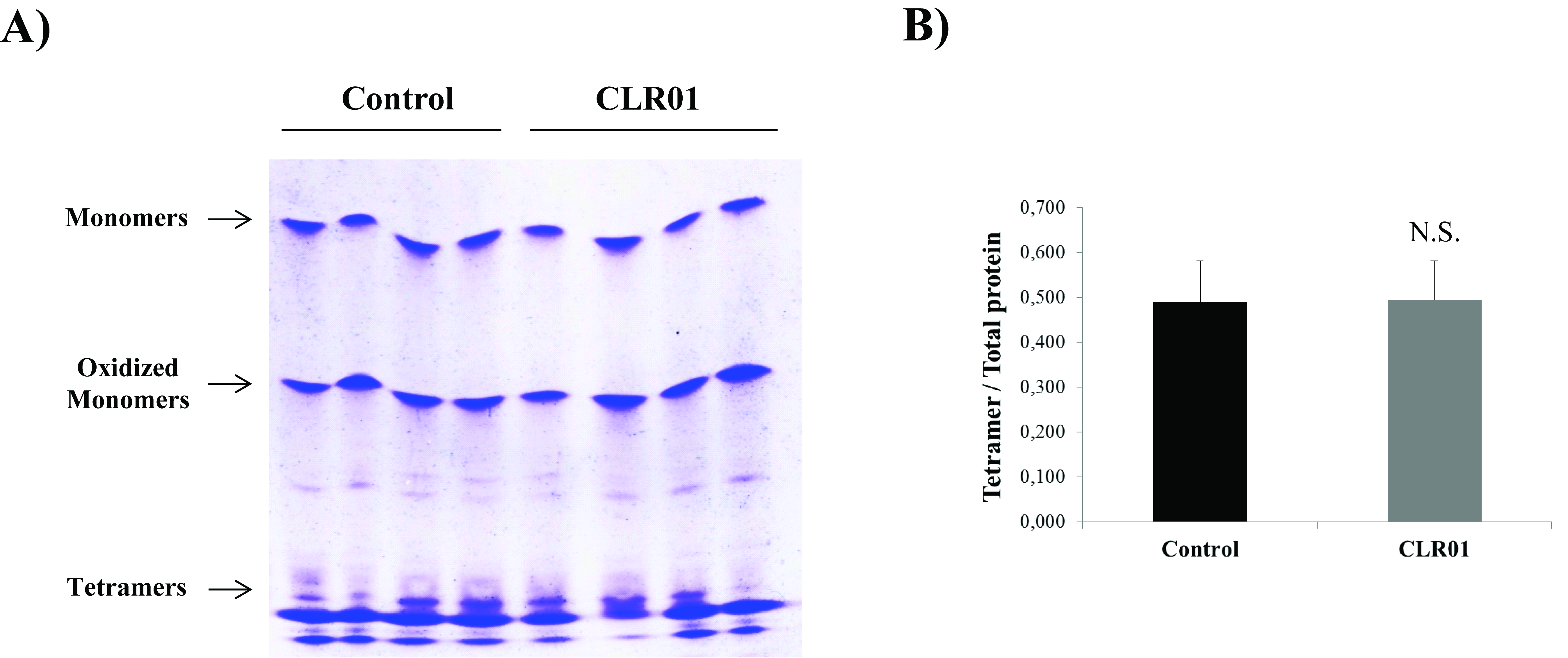

Supplement: Supplementary file 7 — High resolution image (TIFF 4301 kb) [file 13311_2013_256_MOESM4_ESM.tif]
